# Telecentric stereo 3D imaging with isotropic micrometer resolution bridges macro- and microscale in small Lepidopterans

**DOI:** 10.1038/s41598-025-13795-6

**Published:** 2025-08-06

**Authors:** Andreas Walter Stark, Adeoluwa Osadare, Matthew Guo, Gregor Joerg Gentsch, Dennis Boettger, Gunnar Brehm, Christian Franke

**Affiliations:** 1 Institute of Applied Optics and Biophysics, Friedrich Schiller University Jena, 07741 Jena, Germany; 2 Phyletisches Museum, Friedrich Schiller University Jena, 07743 Jena, Germany; 3 Jena Center for Soft Matter, Friedrich Schiller University Jena, 07743 Jena, Germany; 4 Abbe Center of Photonics, Friedrich Schiller University Jena, 07743 Jena, Germany

**Keywords:** Biological imaging, Structured illumination, Stereo photogrammetry, Optical sensors, 3-D reconstruction, Animal physiology

## Abstract

We present a straightforward, application-driven telecentric stereo 3D-measurement system for high-precision measurements, designed for applications ranging from industrial quality control to biological research including scanning of Lepidoptera moths. Utilizing a dual-camera setup with telecentric lenses and structured illumination, our system achieves lateral resolution of 8.0 $\upmu$m and axial resolution of 4.46 $\upmu$m in a measurement volume of 11 mm $\times 11$ mm $\times 6$ mm. We address challenges typically encountered when using standard libraries like OpenCV, e.g. in extrinsic parameter estimation using a dedicated calibration method that corrects for a potential model mismatch due to telecentricity. Our approach adapts existing methods, such as telecentric stereo vision and structured illumination, into an optimized, user-friendly system tailored for life science research, enabling detailed 3D-reconstructions of scattering objects, such as small moths, with isotropic micrometer accuracy. This work presents an application-driven approach for biological 3D-metrology by integrating existing technologies (telecentric stereo vision, structured illumination) into a specialized imaging platform suitable for non-invasive morphological studies. Unlike conventional CT or microscopic approaches, our method provides a balance of precision, scalability, and practical usability for non-expert users with the aim to study developmental changes in species under varying environmental conditions, while also methodically bridging the gap between macroscopic and microscopic resolution in biological imaging.

## Introduction

Accurate three-dimensional (3D) imaging of biological specimens is essential for revealing how organisms sense, move, and adapt to their environment, be it through the fine whiskers of rodents^1,2^ or the fragile components of insects^3,4^, like wings of moths (Lepidoptera)^5,6^. These intricate structures are often soft, light-scattering, and easily deformed, requiring non-contact measurement techniques to capture their morphology without causing damage. The accurate 3D-measurement of biological specimens, such as small rodents^1,2^ and insects^3^, e.g. moths (Lepidoptera)^5^, is critical for understanding how, for example, multisensory integration in mammals’ functions and also how species adapt to environmental changes. Such fine and delicate structures (whiskers, wings, etc.) should be measured with a contactless method, due to them being prone to damage or distortion. These measurements should also be conducted in a reasonable time frame, allowing for statistically relevant sample sizes. Traditional (stereo-) photogrammetric methods are typically limited to lateral resolutions above 50 $\upmu$m (laterally)^7–10^—although some can reach a resolution around 10 $\upmu$m^11,12^, while microscopic techniques, though offering sub-micrometer resolution, are constrained by a small field of view—often not exceeding 2 mm. For instance, confocal microscopy typically provides a lateral resolutions in the range of 0.2–1.0 $\upmu$m but is limited to volumes $<$ $1$ mm$^3$. Computer tomographic (CT) methods, while satisfying most conditions, are time and resource-intensive for larger studies and often lack the sensitivity to resolve subtle biological structures^13^, even though the achieved resolution can reach 10 $\upmu$m isotropically. While there have been telecentric approaches to 3D-measurement, allowing principally a high resolution and still a macroscopic field of view^14–17^, biological applications using standard libraries and structured illumination remain underexplored in this segment of metrology. Some of the mentioned previous studies have already demonstrated the value of telecentric lenses in structured light systems, particularly in industrial metrology applications where high depth precision and minimal perspective distortion are essential^14,17^. However, these setups often require complex calibration procedures, custom calibration targets, or precise mechanical alignment, which may hinder their applicability in biological laboratories or for non-experts in optical metrology in general. Our approach builds on the mentioned ideas and the approached from endocentric 3D-measurement with structured light^7,8,11^, aiming to reduce technical barriers while maintaining micrometer-scale accuracy, thereby opening the field for non-expert users in biology and related disciplines. Here, we aim to lower the entry barrier for life scientists to utilize high-quality 3D-measurements, opening up new avenues of quantification. In this study, we introduce a telecentric stereo 3D-measurement system that overcomes these limitations. While avoiding problems of standard open-source libraries such as OpenCV^7,8^ that are designed for pinhole-based camera models, we provide an in principle sub-cellular, isotropic resolution suitable for applications in both macro- and microscopic domains. We show that telecentric stereo-imaging, in combination with structured illumination, enables precise volumetric reconstructions with isotropic single digit micrometer resolution. To achieve this, we have adapted existing methodologies and provide a streamlined measurement system for biological research, reducing the complexity of existing optical metrology techniques. Finally, we demonstrate the system’s applicability in biological questions through the 3D-measurement of a small moth specimen (Geometridae: Idaea sp.), capturing subtle details that could e.g. provide insights into morphological adaptations of insect species along environmental gradients^5^. Small Lepidopterans exhibit fragile, complex wing structures and subtle morphological variations, making them an ideal test case for high-precision, non-contact volume measurement. Unlike CT, which requires extensive resources, preparation and measurement time, and confocal microscopy, which has limited field of view, our method allows quick, high-resolution scanning of macroscopical organisms with minimal setup effort. The measurement volume of 11 mm $\times 11$ mm $\times 6$ mm together with a nearly cubic isotropic resolution effectively bridges the macroscopic and the microscopic scale.

## Results

### Reconstruction of Lepidopterans

**Fig. 1:**
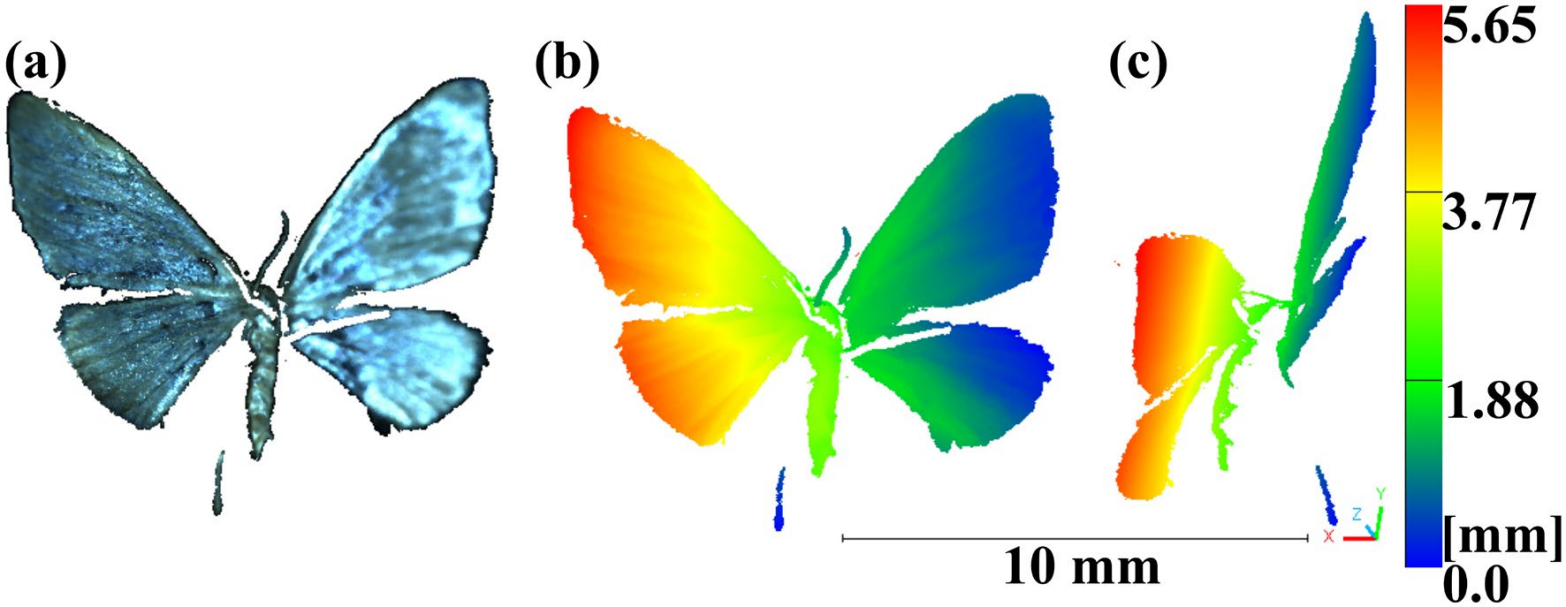
(**a**) 3D-reconstruction of a small moth species (Idaea sp.) in front view and real colors, (**b**) front view with z coordinates as color, (**c**) side view with z coordinates as color. Scale bar and color bar in mm. Images were acquired using proprietary acquisition software developed by the authors, based in part on Allied Vision’s Vimba SDK (AVT SDK download), and processed with CloudCompare v2.12 (www.cloudcompare.org). Point clouds are available in the supplementary file^19^.

Within this work a telecentric 3D-measurement setup with active, structured illumination was realized and adapted. The measurement volume had dimensions of 11 mm $\times 11$ mm $\times 6$ mm and allowed a lateral resolution of 8 $\upmu$m and a precision of 4.46 $\upmu$m in axial direction. To demonstrate the applicability and sensibility of this high-precision 3D-measurement method to biological samples, we measured a moth specimen (Geometridae: Idaea sp.) with the system. This specific sample is of interest due to its delicate nature, its fine details and the morphological adaptations of moths that occur as a reaction to different living conditions. The full set of raw measurement data generated during the current study is available via Zenodo^18^, including the complete image sequences used for 3D-reconstruction. An exemplary visualization of one of the reconstructions can be seen in Fig. 1.

**Fig. 2:**
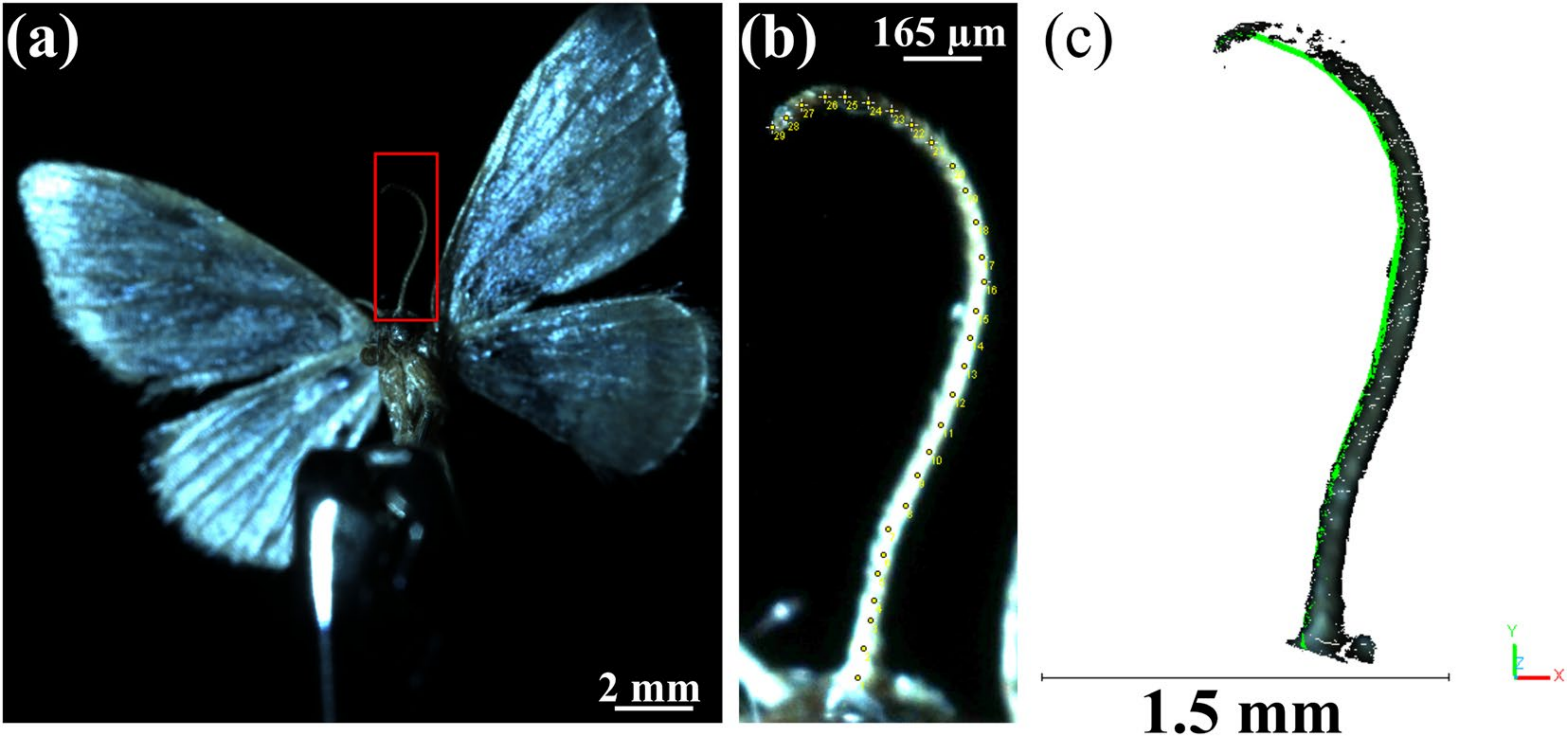
(**a**) Single image with homogeneous illumination from the first camera of the same moth as in Fig. 1 but under a slightly different angle. The image part containing the antennae of the moth is outlined in red. (**b**) Cropped image part containing the antennae increased in brightness to improve visibility. Comparable to^4^ a 2D-measurement of the length of the antennae was performed using Fiji/imageJ. The resulting length (each pixel representing 4 $\upmu$m) was calculated to 2.576 mm. (**c**) Crop of the 3D-reconstruction of the moth under this view, containing the antennae. Using CloudCompare a polyline was fitted on the point cloud. The length of the polyline and the points representing the antennae accordingly was measured to be 3.733 mm. Images were acquired using software based on Allied Vision’s Vimba SDK (AVT SDK download) and processed using Fiji (ImageJ v1.54, ImageJ download) and CloudCompare v2.12 (www.cloudcompare.org). Point clouds are available in the Supplementary file^19^.

Figure 1a shows the true-colour point cloud, Fig. 1b the same data colour-coded by depth, and Fig. 1c provides a side view to visualise the overall body curvature. Even though the tiny specimen had a length of below 4.4 mm, a maximum width of 0.75 mm and a wingspan of 10.75 mm, Fig. 1 showcases several distinct features of the moth, reconstructed in high detail. The measurement procedure was repeated several times for different positions, the resulting point clouds are available in the supplementary file^19^. To give an example of the advantage of this 3D-measurement techniques of small specimen as compared to measurement in 2D^4^ we look into the images and the reconstruction of an antennae of the moth (see Fig. 2). Here we show the full view of the first camera under homogeneous illumination (Fig. 2a) and enlarged the antennae with measurement spots in 2D, to retrieve the geometrical length as in^4^ (Fig. 2b). We then used CloudCompare^20^ to fit a polyline on the reconstruction of the antennae, stemming from the 3D-measurement in this position (Fig. 2c).

A comparison between the 2D-image and the 3D-reconstruction of the moth’s antennae revealed a significant discrepancy in the measured length: while the 2D-projection suggests a length of approximately 2.576 mm, the corresponding 3D-polyline measures around 3.733 mm. This difference arises from the inherent curvature of the antennae in three-dimensional space, which is not captured in the 2D image due to the foreshortening effect introduced by the projection. Consequently, relying on 2D-measurements alone would result in a substantial underestimation of true anatomical dimensions. This point cloud data can enable the detailed quantification of features at the micrometer scale, as well as the precise comparison of different phenotypes or species. To date, we only show single-view, i.e. incomplete, reconstructions of this moth as proof-of-concept. Nevertheless, these single view measurements were repeated for different distinct positions of the moth (Figs. 1 and 2), already suggesting the possibility for a completed 360$^\circ$-3D-reconstruction. 13 of such created point clouds—including the two shown here—can be found in the online repository^19^ for reference and further use.

Given the rather short field-of-depth of the system, the repositioning of the tiny specimen and subsequent point-cloud alignment is extremely delicate and will be the subject of future work. In turn, this would open up quantitative volumetric measurements of different specimen, e.g. from different habitat zones with morphological adaptations or other phenotypic classes^5^. Additionally, further improvements in the structured illumination approach could enhance the resolution towards even finer details. The presented custom calibration successfully corrected issues encountered with standard libraries, enabling accurate determination of the extrinsic parameters. Our future work will focus on optimizing the system by adapting the setup for a robust 360$^\circ$-reconstruction from consecutive single-view 3D-point clouds. The compact and straightforward nature of the current setup could also enable its field deployment and thus extending the approach to other biological specimens.

### Reconstruction of reference objects of known dimensions

With this framework different objects, such as a commonly used reference plane with a surface roughness < 1 $\upmu$m, a microscope alignment plate with a millimeter scaling 3a and a 3D-printed resin sphere could be properly reconstructed (see Fig. 8b).

**Fig. 3:**
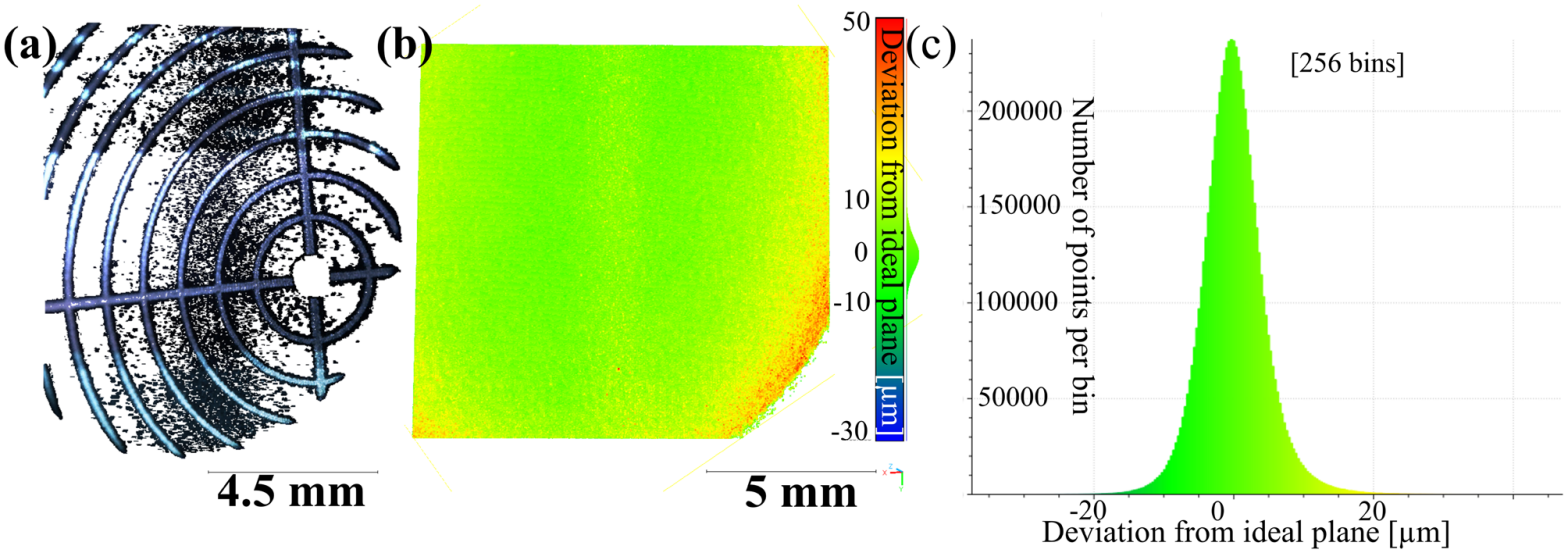
(**a**) 3D-reconstruction of lens mount alignment plate (see Fig. 6, Thorlabs, LMR1AP, 1 mm spacing between circles) used to estimate scale. Please note that the goal of the reconstruction was to find the bright lines not the strongly absorbing dark background. We were able to reconstruct some of those points as well, showcasing well-aligned reconstruction results even for uncooperative surfaces. (**b**) 3D-reconstruction of reference plane with a roughness < 1 $\upmu$m. Here the entire field of view has been shown in a color scale, that represents the distance of each point from the best fit plane. The deviation increases slightly to the borders of the measurement field, as known from photogrammetry. The missing part in the lower left can be explained with a slight miss alignment of illumination and field of view. (**c**) Histogram of the plane reconstruction, showcasing how the standard deviation is formed from these results. Point clouds were processed with CloudCompare v2.12 (www.cloudcompare.org) and are available in the Supplementary file^19^.

We used the point cloud of the alignment plate (Fig. 3a, 6b) to calculate the scaling of the system in real world coordinates, resulting in a rescaling factor of 1.9928. The system’s performance was validated using the calibrated reference plane (Fig. 3b), achieving a standard deviation of 4.46 $\upmu$m (Fig. 3c) for the full field of view while only removing outliers with a correlation value^7,8,11,21^ below 0.9. All reconstructions can be found in the Supplementary file^19^.

To showcase the 3D-precision an additional measurements with a calibrated, metrology-grade standard spheres (radius of 5 mm, maximum deviation from ideal sphere shape 1.6 $\upmu$m) have been conducted. The deviation from the ideal sphere shape for all reconstructed points and the central 48.5 $\%$ are shown in 4.

**Fig. 4:**
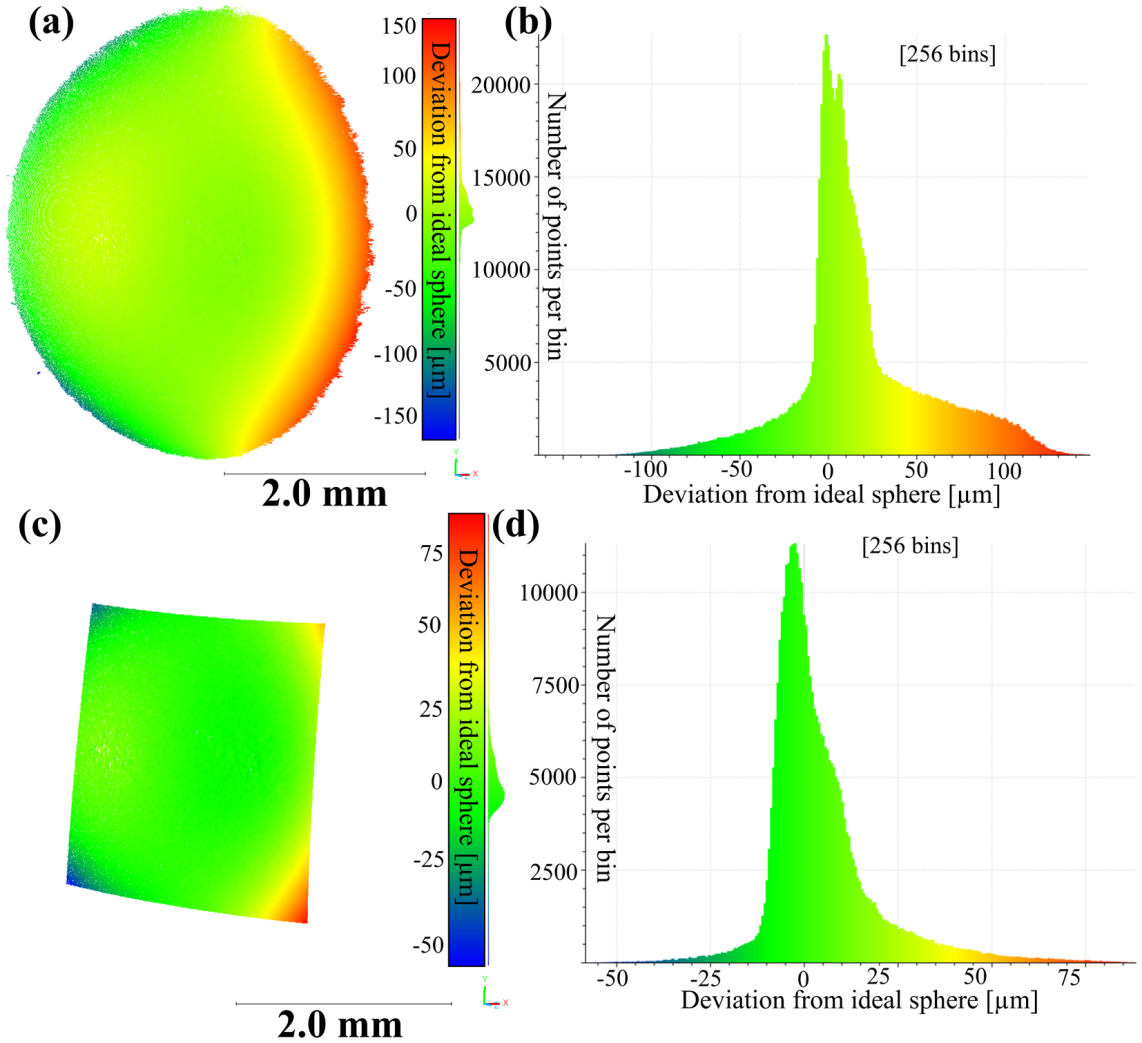
(**a**) Differences of individual 3D-points from best fit sphere for every trustworthy reconstructed point (threshold for correlation was set to 0.9). Here a metrology-grade standard spheres (radius of 5 mm, maximum deviation from ideal sphere shape 1.6 $\upmu$m) was used. (**b**) Histogram of error distribution. The standard deviation for the reconstruction of all trustworthy points was found to be 26.0 $\upmu$m. (**c**) Differences of individual 3D-points from best fit sphere for central 18.5 % reconstructed points (threshold for correlation was set to 0.9). (**d**) Histogram of the error distribution of the central 48.5 % of points. The standard deviation is 8.0 $\upmu$m for this distribution. Point clouds were processed with CloudCompare v2.12 (www.cloudcompare.org) and are available in the supplementary file^19^.

The high-precision reference measurement of a metrology-grade calibration sphere (Fig. 4) revealed a good reconstruction quality, indicated by the standard deviation from an ideal sphere of 26.0 $\upmu$m. Notably the deviations from a perfect sphere shape are increasing to the sides of the reconstruction. This is due to the relative slope of the surface to the viewing direction. The higher this slop, the higher is the error of reconstruction. As a result, for a central patch of the reconstruction - here $48.5 \%$ with a weaker slope to the viewing direction the standard deviation decreases to 8.0 $\upmu$m. Given the optical quality of the telecentric lenses used, such distortion is typically negligible and often not corrected in comparable systems^14,16,17^.

While distortion correction would be possible in principle, it requires complex modeling and calibration steps^15^ that would significantly increase the entry barrier for non-expert users. In line with the overall design goal of providing a low-threshold, application-oriented 3D-measurement system, we consider the omission of distortion correction to be a deliberate and acceptable trade-off between usability and precision.

### Comparison of telecentric stereo calibration methods, effort and precision vs low entrance barrier

To contextualize our approach, we compared our measurement strategy to existing methods from the literature (Table 1). Many previously published approaches for telecentric stereo systems omit full distortion correction and instead rely on simplified intrinsic models with 3 $\times$ 3 matrices and diagonal focal length entries^14,16,17^. This reflects the fact that image distortion is typically very low for high-quality telecentric lenses, making such corrections negligible in many practical applications.

**Table 1:** Comparison of measurement strategies for telecentric stereo vision systems regarding required calibration, availability of code, resolution and impact of distortion. Please note than some previous publications did not mention the lateral resolution of the system in use, therefore a number for this resolution could not provided.

Reference	Intrinsic calib.	Extrinsic calib.	Code access	Resolution	Distortion
This work	3 $\times$ 3 matrix, no distortion correction	From real 3D-object correlations	Yes	8 $\upmu$m lateral; 4.5 $\upmu$m axial	No residuals found
Chen et al. (2022)^17^	3 $\times$ 3 matrix, no distortion correction	Known-depth custom object	No	lateral: not specified; $\sim$ $20$ $\mu m$ axial; $\sim$1 $\mu m$ reproducibilty;	No residuals reported
Hu et al. (2021)^15^	3 $\times$ 3 matrix and distortion correction	Checkerboard with constraints	No	8.27 $\mu m$ lateral; $\sim$ $1$ $\upmu$m reprojection error	No residuals reported
Zhang et al. (2019)^14^	3 $\times$ 3 matrix, no distortion correction	3D-printed calibration object	No	lateral: not specified; $\sim$ $20$ $\mu m$ axial	No residuals reported
Liu et al. (2016)^16^	3 $\times$ 3 matrix, no distortion correction	Checkerboard-derived epipolar calibration	No	lateral: not specified; $\sim 10 \upmu$m axial for control points	No residuals reported

Our system follows this pragmatic approach and forgoes distortion correction in the intrinsic calibration. The extrinsic parameters are obtained from measurements of real 3D-objects, using robust correlation-based matching instead of reference targets like checkerboards or precisely manufactured calibration objects. This reduces the setup and calibration complexity, which is particularly beneficial for interdisciplinary users or applications in life sciences, where calibration targets might be impractical or unavailable.

Most comparable systems report no visible distortion effects and also we did not observe depth-dependent residuals. The implementation of distortion correction would require additional modeling^15^ and expert knowledge, raising the entry barrier for non-specialist users. Therefore, we argue that our method offers a favorable trade-off between simplicity and achievable precision, in line with most published approaches, while allowing for robust micrometer-scale reconstruction across extended depth ranges.

## Discussion

In conclusion, we have set up and adapted a telecentric stereo 3D-measurement system that offers a unique combination of high resolution and large measurement volume when applied to biological photogrammetry, enabling precise volumetric analysis of specimens. Potential sources of error include subpixel inaccuracies in correspondence detection, 3D-printing deviations of the reference sphere, and the delicate positioning of biological specimens. Future improvements will include automated alignment procedures and statistical analysis of volumetric precision to further quantify the measurement uncertainty. Compared to conventional stereophotogrammetric setups, our telecentric system already achieved an isotropic single digit micrometer resolution, i.e. in principle in the sub-cellular range, without additional post-processing. This is in good agreement with state-of-the-art telecentric stereo systems, but its use for biological specimens is novel. Our method, validated through the 3D-reconstruction of reference objects and specimen, demonstrates the potential for applications in developmental biology and other fields, where accurate, quantitative 3D-data is essential. This work paves the way for future advancements in combining macroscopic and microscopic imaging techniques, offering a new perspective on the study of morphological phenotypes in various species and potentially a plethora of adjunct fields of study.

## Methods

### Workflow summary

**Fig. 5:**
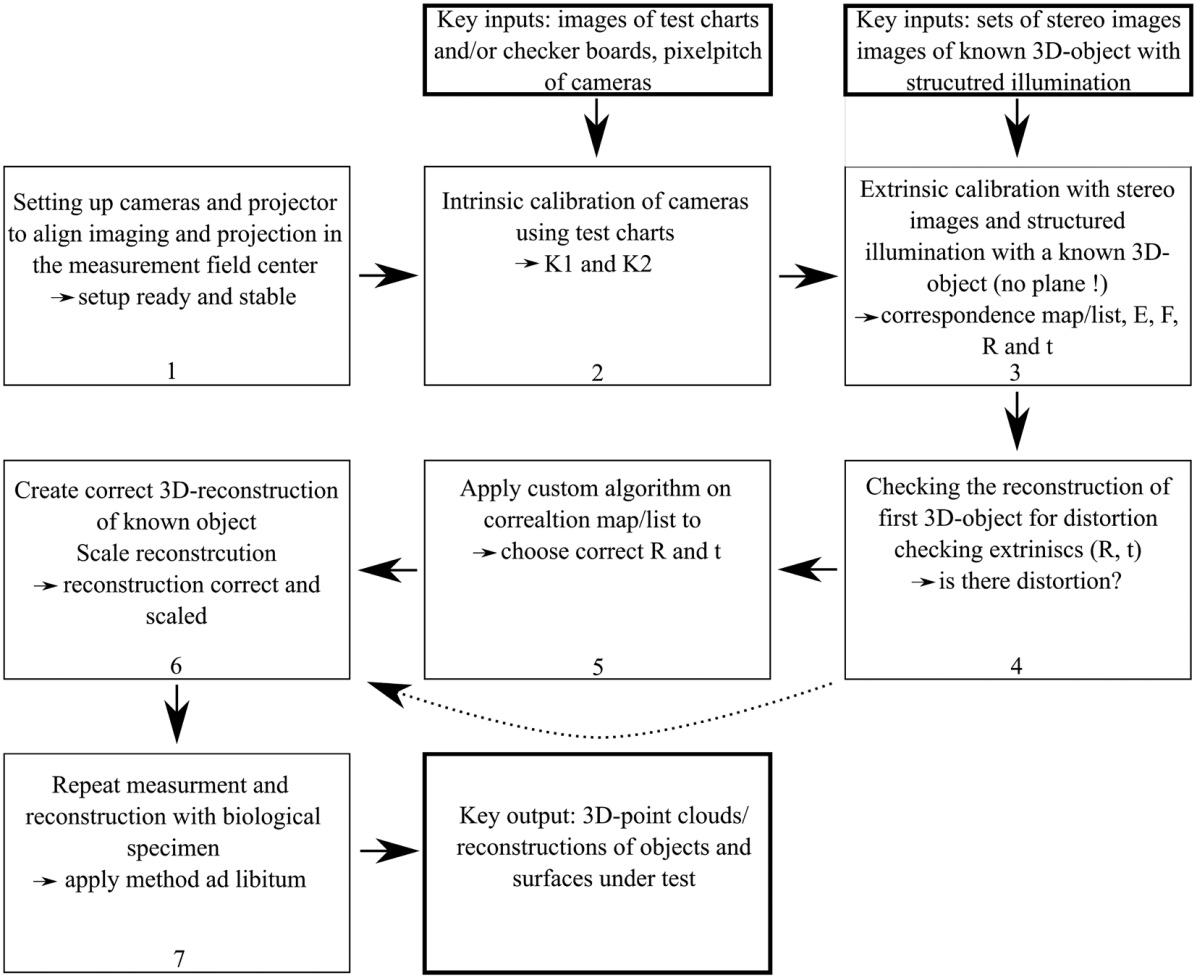
Flowchart representing the steps necessary to build and if needed correct the 3D-reconstruction methods applied here. Key inputs and outputs are indicated by bolt boxes. Please note that step 5 can be skipped if the reconstruction is undistorted after step 4.

The methodological workflow for the telecentric stereo 3D-measurement process is illustrated in Fig. 5. It consists of the following main steps:

1. System setup: The cameras and structured light projector are mechanically aligned such that the projection axes and the optical axes of both telecentric lenses intersect at the center of the intended measurement volume. This ensures a physically stable and well-defined overlap region. Needed time: 1 to 4 h, depending on user experience (DOUE).
2. Intrinsic calibration: Using microscopic test charts and grid targets, the intrinsic parameters of both cameras (matrices $K_1$ and $K_2$) are estimated to account for magnification and lateral resolution. Needed time: 1 to 4 h (DOUE).
3. Extrinsic calibration—initial step: Stereo image sequences of a known 3D-reference object (e.g., a resin-printed sphere) under structured illumination are captured. Based on subpixel-accurate correlation, an initial set of extrinsic parameters (rotation *R* and translation *t*) is derived using standard procedures (e.g., from the essential and fundamental matrix). Needed time: 5 to 10 min measurement time, depending on algorithm seconds^21^ to 30 minutes for the extrinsic calibration.
4. Distortion check: The reconstructed point cloud is compared against the known geometry of the reference object. Significant deviations or warping indicate a model mismatch caused by standard pinhole assumptions. Needed time: 5 to 10 min.
5. Model correction: A custom algorithm is applied to resolve the ambiguity introduced by mismatched assumptions in the extrinsic calibration. With the help of the algorithm (and this manuscript) the user can identify the correct combination of *R* and *t* that matches the telecentric projection geometry. Please note that this step can be skipped if no deviations are found. 2 min to 30 min (DOUE).
6. Final 3D-reconstruction and scaling: The corrected extrinsic parameters are used to generate an accurate, undistorted point cloud. Scaling is performed using features with known real-world dimensions, enabling quantitative interpretation. Needed time: 30 s for each reconstruction.
7. Application to biological specimens: Once validated, the system is applied to fragile biological samples. Multiple measurements may be repeated at different orientations to allow for full 3D-reconstruction or volumetric analysis. Depending on sample properties and experience level of user 5 to 20 min per sample and reconstruction.

### Setting up a telecentric stereo system with structured illumination I: optical system setup

Photogrammetric 3D-measurement techniques with structured illumination are state of the art for a lot of applications. Also the more specialized sub-methodology—using telecentric cameras instead of ‘regular’ or endocentric cameras—has been applied successfully also with structured illumination. However, these instances have been performed by experts of image processing and optical metrology. Researchers from other fields with limited experience in optical metrology may benefit from a simplified, pre-configured system. Here, we will try to explain and to guide through the steps necessary to setup and reproduce our experiment and point out problems, that we ran into. The first part is assembling the mechanical setup: Our advice is, to start with the projector and one camera. Here Allied Vision (Stadtroda, Germany) Alvium cameras (1800 U-811c color C-Mount), with 2848 $\times$ 2848 pixels each and a pixel pitch of 2.74 $\upmu$m have been used. The telecentric lenses were of the type LM1123TC from Kowa (2/3” 0.69–0.88x) and a low cost off-the-shelf projector (Technaxx Mini LED Beamer TX-113) was in use. The projector has to be connected to the same computer, the cameras are connected to. Also, at least two additional optics, for example lens doublets with 2” diameter and a focal length of 75 mm (in this case Thorlabs AC508-075-A-ML) can be used to minimize the field of projection to the desired size of 10 mm $\times$ 10 mm. A blank piece of white paper can be used as a screen. One of the cameras (with a telecentric objective) can be used here to evaluate contrast, field of view and depth of field. The working distance with the system in use in this case was 12 cm. We experienced that an almost completely closed aperture on the imaging as well as on the projection side yields the best depth of field and depth of projection—but also requires significantly more imaging time of up to a few seconds per images/images pair for the biological sample. However, these measurements created the best images and also the best 3D-reconstruction. When the center of projection and the center of imaging of the first camera are overlapping, the second camera can be added to image the same center—the center of the measurement field (see Fig. 7a). It might be of interest to remark, that the achievable 3D-precision is proportional to the sine of the stereo angle of the system. A larger angle also reduces the overlap of the fields of view and increases the risk of occlusions within the measurement volume. Therefore a sweet spot for each application has to be found. Here we realized an angle of approximately 38.5$^\circ$ and a baseline length of approximately 9 cm. After the physical setup the system has to be calibrated, namely intrinsically, so in terms of imaging process by each camera, and extrinsically, in terms of the position of the cameras towards each other.

### Setting up a telecentric stereo system with structured illumination II: system calibration

The imaging process by common or endocentric lenses in first order approximation can be mathematically described with the pinhole model, yielding in a 3 $\times$ 3 matrix ($K_{Endo}$) with the focal length *f* in the spatial directions *x* and *y*, as well as the projected position of the pinhole in the camera plane with the coordinates ($m_x$,$m_y$) (see Eq. (1)). Also, a shearing parameter *s* can be added to accommodate an angle between the pixel orientation in the lateral spatial directions. In comparison, the imaging process with a telecentric lens, as used in this study, can be described with a matrix $K_{Tele}$ that has nonzero values only on the main diagonal (see Eq. 2). These are the magnification divided by the pixel pitch in *x* and *y* and a 1^14–16^.

1 $$\begin{aligned} K_{Endo}= & \begin{bmatrix} f_{x} & s & m_{x} \\ 0 & f_{y} & m_{y} \\ 0 & 0 & 1 \end{bmatrix} \end{aligned}$$

2 $$\begin{aligned} K_{Tele}= & \begin{bmatrix} \frac{M_x}{pp} & 0 & 0 \\ 0 & \frac{M_y}{pp} & 0 \\ 0 & 0 & 1 \end{bmatrix} \end{aligned}$$

Mathematically, this difference resembles the idea that the viewing rays of endocentric cameras are meeting in exactly one point in space, the pinhole. On the other hand telecentric imaging resembles a parallel projection, so that the meeting point of the parallel viewing rays lies at infinity. Like in all 3D-measurement approaches, precise calibration of the system, i.e. the determination of *K*, results in high accuracy and precision, crucial for reconstructing delicate structures like moth bodies. Especially in the presented case, i.e. the rededication of a known method and open-source libraries to a customized telecentric imaging system, the calibration is crucial.

**Fig. 6:**
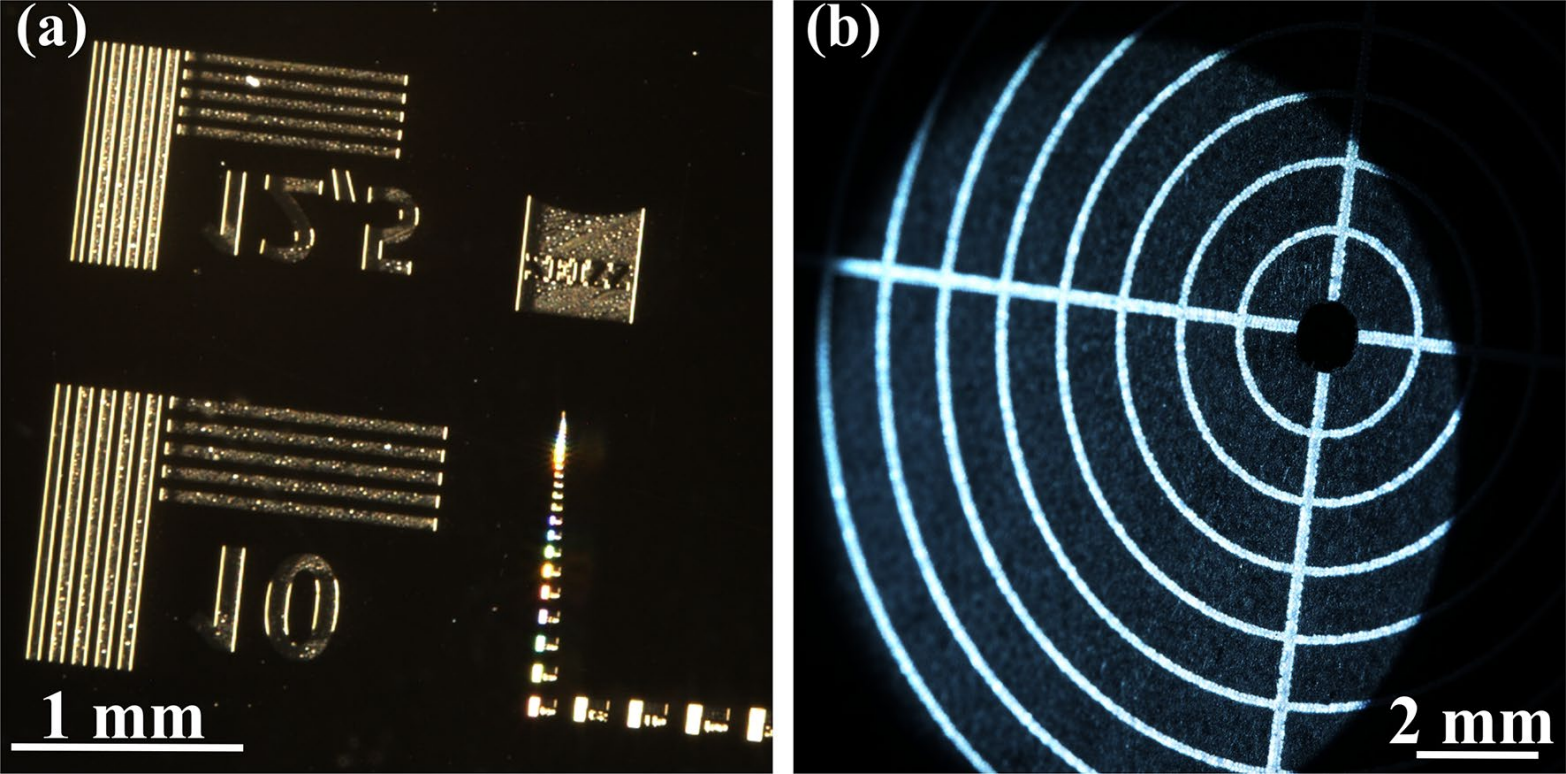
(**a**) Image (cropped) of microscopic reference chart used to estimate the lateral resolution and magnification. (**b**) Images of a lens mount alignment plate (Thorlabs, LMR1AP, 1 mm spacing between circles, left) used to estimate scale. Images were acquired using software based on Allied Vision’s Vimba SDK (AVT SDK download). The complete images are available in the supplementary file^19^.

The first part of calibration in photogrammetry is finding an appropriate imaging matrix according to Eq. (2) for each camera. To intrinsically calibrate, marked reference planes (graph paper and checker boards as well as a microscopic test chart, see Fig. 6) have been used to identify the magnification and the lateral resolution by comparing real size of objects to imaged size of objects^14–16^ (see Supplementary file^19^). For non-expert users a simplified version of finding the magnification could be using graph paper and taking images of it when orientated perpendicular to the viewing direction of one of the cameras and then repeating this for the second camera. With the knowledge of the pixel pitch a comparison of the length of the grid marks in pixels (multiplied with the pixel pitch) can be compared with the real size and such found an approximation for the magnification in *x* and *y*. Please note that this simplified method might be imprecise but was found to deliver comparable results to more sophisticated approaches by the authors of this manuscript.

The pixel pitch has been taken from the camera manufacturer. We found the effective magnification of the lenses to be 1.46 and thus the lateral resolution to be 8 $\upmu$m (using 6a). Here, we took the size of the reference structure in the images in pixel into account and evaluated the resolution from the visibility of the smallest resolved structure. The intrinsic matrices $K_1$ and $K_2$ for both cameras were found and are presented in Eq. (3) and (4).

3 $$\begin{aligned} K_{1}= & \begin{bmatrix} 252,000 & 0 & 0 \\ 0 & 252,000 & 0 \\ 0 & 0 & 1 \end{bmatrix} \end{aligned}$$

4 $$\begin{aligned} K_{2}= & \begin{bmatrix} 251,800 & 0 & 0 \\ 0 & 253,000 & 0 \\ 0 & 0 & 1 \end{bmatrix} \end{aligned}$$

The alignment plate (Fig. 6b) on the other hand, was later used to retrieve the real world scale from the setup. Please note that this was done after the 3D-reconstruction of the alignment plate, also to support the reliability of the system.

**Fig. 7:**
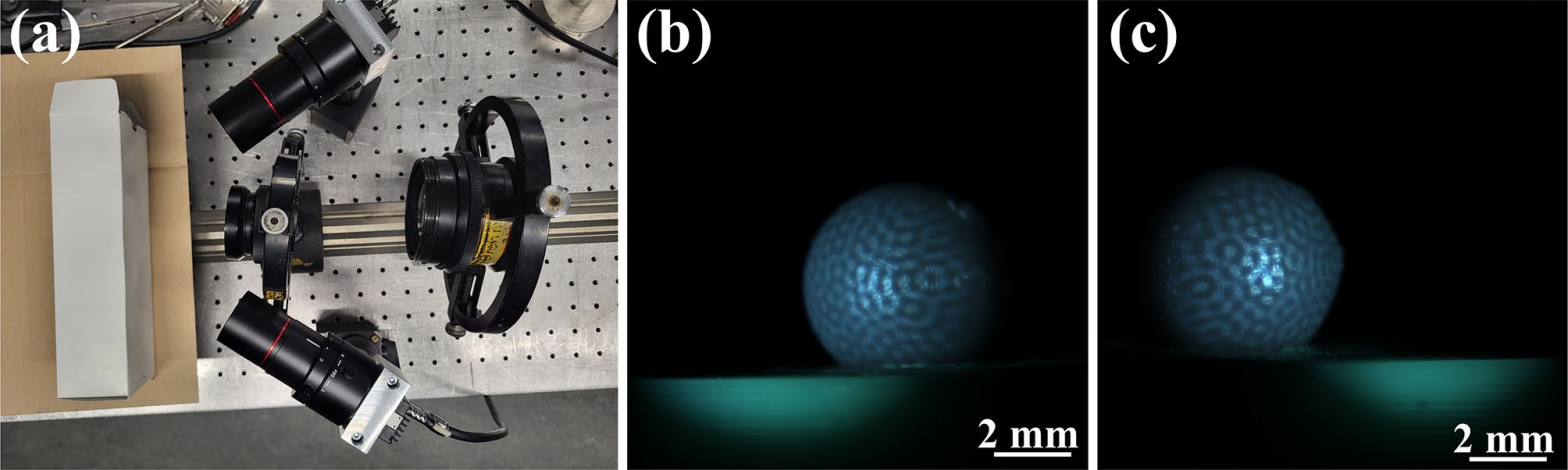
Measurement setup (**a**), with two cameras with telecentric lenses and two projecting lenses (projector outside of image). Photograph taken by the authors using a consumer smartphone camera. (**b**) 3D-printed sphere (radius = 2.5 mm) as imaged from first and (**c**) second camera under structured illumination (Supplementary file^19^). Images were acquired using software based on Allied Vision’s Vimba SDK (AVT SDK download).

The straightforward 3D-measurement setup (see Fig. 7a) is comprised of the two mentioned cameras and a commercial projector with a magnifying lens assembly to fill a 11 mm $\times 11$ mm $\times 6$ mm measurement volume. The second step is to find the extrinsic parameters (extrinsics), i.e. the rotation and translation from the first image plane (camera 1) to the second one (camera 2). Here, a measurement of a resin printed sphere with a radius of 2.5 mm has been taken with 100 stereo images and varying structured illumination, comparable to already published studies^7,8,11^, see Fig. 7b,c. A recent, openly accessible reconstruction algorithm can be found here^21^. This rapidly fast algorithm might be difficult to understand and implement for non-expert users.

### Correcting the wrong extrinsic calibration and scaling

**Fig. 8:**
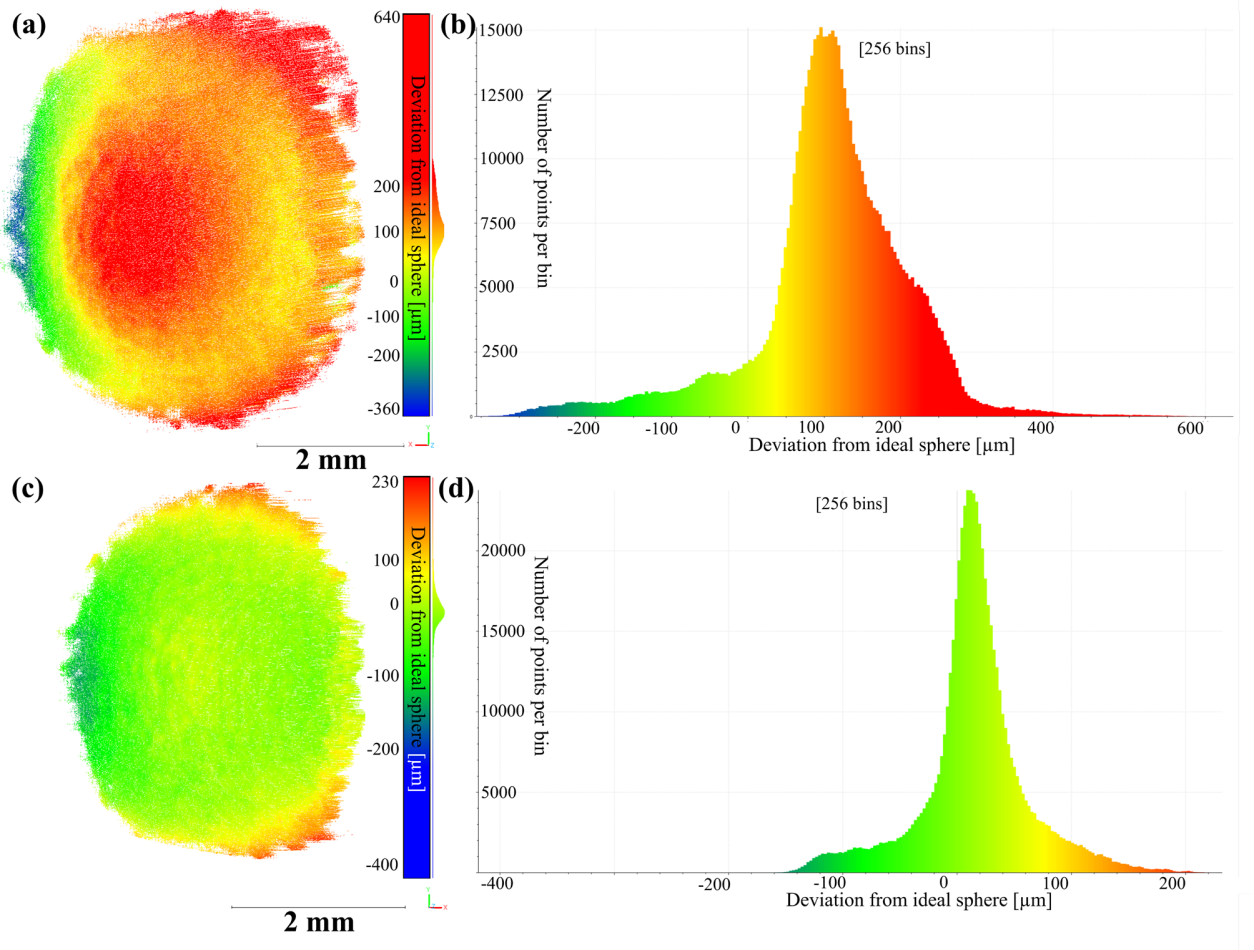
(**a**) Distorted and unscaled point cloud/3D-reconstruction of the resin sphere (see Fig. 7b,c). The differences of each point to a best fit sphere are shown in false colors. (**b**) Histogram of the distribution of distances of the unscaled and distorted reconstruction of the resin sphere. Please note that the color scale has been adjusted to match the one for the undistorted reconstruction for visibility reasons. (**c**) Undistorted and scaled point cloud/3D-reconstruction of the resin sphere (see Fig. 7b,c). The differences of each point to a best fit sphere are shown in false colors. (**d**) Histogram of the distribution of distances of the undistorted and scaled reconstruction of the resin sphere. The data was rendered in CloudCompare^20^. Points from the holding platform (Fig. 7b,c) have been removed. *x*, *y* and *z* -coordinates shown in tripod lower right corner. Point clouds were processed with CloudCompare v2.12 (www.cloudcompare.org) and are available in supplementary file^19^.

Using the temporal correlation search for correspondence pairs^7,8,11^ yielded sub-pixel accurate results. However, using these results to calculate the initial extrinsic parameters of the measurement system resulted in heavily distorted 3D-reconstructions when compared to a best fit sphere (see Fig. 8a).

The deviation in this case were obvious due to the vast misalignment of the reconstruction with a best fit sphere, indicated by the color distributions in Fig. 8. We also encountered distorted reconstruction when putting other samples under test. In this context we present only the sphere, as a clear reference for a misreconstruction model. Non-expert users can however take other objects of known (relative) dimensions to check for a correct reconstruction. In some cases, the standard calibration (step 4) may already yield the correct extrinsic parameters. In such situations, step 5 can be skipped, as no deviation is observed in the initial 3D-reconstruction. The significant errors encountered by us is not surprising, as standard libraries that we aim to utilize to enable open-source access and low entry barriers for non-experts such as life-scientists, e.g. OpenCV, presume non-telecentric/endocentric imaging systems that can be described by a pinhole model. Thus, they prove to be inadequate if applied naively on the extrinsic calibration, leading to distorted reconstructions (Fig. 8a). In mathematical terms, a set of two rotational matrices and two translation vectors is found when calibrating extrinsically this way. However, due to the inherent model mismatch, the algorithm will produce likewise mismatched extrinsics by discriminating the wrong set of matrix and vector. To address this, we rewrote a custom algorithm inspired by standard literature of the field^22^ that allowed to correctly choose from the possible set of extrinsics to accommodate the model mismatch (compare Eq. (1), (2) and see the Supplementary file^19^). Utilizing the correct extrinsic parameters consistent with the telecentric model, the identified pixel-pair correspondences were then straightforwardly used to reconstruct undistorted point clouds as in^7,8,11^ via triangulation. Please note that the two possible translation vectors only differ by sign while the two options for the rotational matrix represent a real second perspective and the reprojection in the first camera view. In principle it is not important whether ‘*t*’ or ‘$-t$’ are chosen. This creates reconstructions with a sign change on the *x* and *z* axes but are geometrically correct. Choosing the wrong rotational matrix on the other hand leads to the described deviation. As a rule of thumb (for non-expert users)—the rotational matrix that shows greater deviation from the identity matrix is (usually) correct. The two rotational matrices for this setup, the incorrect one $R_{incorrect}$ and the correct one $R_{incorrect}$, are shown in Eq. (5) and (6), respectively.

5 $$R_{{incorrect}} = \left[ {\begin{array}{*{20}c} {0.99868} & {0.049236} & {0.014582} \\ { - 0.048842} & {0.99846} & { - 0.026233} \\ { - 0.015851} & {0.025486} & {0.99955} \\ \end{array} } \right]$$

6 $$R_{{correct}} = \left[ {{\text{ }}\begin{array}{*{20}c} { - 0.64667} & { - 0.055855} & { - 0.76073} \\ {0.044468} & { - 0.99838} & {0.035504} \\ { - 0.76148} & { - 0.010869} & {0.6481} \\ \end{array} } \right]$$

The values on the main diagonal of $R_{incorrect}$ (Eq. 5) are close to 1, while all other values are close to 0, coming as a whole close to a unity matrix. On the other hand $R_{correct}$ (Eq. 6) has also values deviation from 1 on the main diagonal and such deviating from 0 aside of it.

The resin sphere was reconstructed using a correlation threshold^7,8^ of 0.3 and removing points from the holding platform. The differences to a best fit-sphere are inidcated by colors in Fig. 8a,c. The strong deviation for the distorted sphere (Fig. 8a) are also present in the histogram (Fig. 8b). After reconstructing with the correct extrinisc parameters (Fig. 8c) for a fixed radius of 2.5 mm a standard deviation from sphere form of 63.1 $\upmu$m was found. The histographic distribution of the corrected reconstruction is shown in Fig. 8d. In addition to measurement noise (see Fig. 4), deviations from an ideal sphere may result from limitations in the manufacturing process of the calibration object itself. As the reference sphere was produced layer by layer via additive manufacturing, slight geometric inaccuracies—such as surface roughness, staircase effects, or warping during cooling—could have contributed to the observed standard deviation. Moreover, residual optical distortions not corrected during calibration may also lead to systematic depth errors, particularly at the sphere’s edges. Note that only a sector of the resin sphere was visible within the overlapping field of view, limiting a fit with a flexible radius to a portion of the full sphere. The actual diameter of the sphere may differ slightly from 5 mm, in addition.

## Supplementary Information

Supplementary Information.

## Data Availability

Data is provided within the manuscript or supplementary information files and are available via figshare: https://doi.org/10.6084/m9.figshare.c.7652423.v1. We have also uploaded the raw data to Zenodo (DOI: 10.5281/zenodo.15432113).

## Code availability

Accession codes: Data underlying the results presented in this paper are available in a supplementary file—separated in 3 sub-files 1, 2 and 3. Here supplementary file 1 includes the images shown in the manuscript, file 2 includes the 3D-point clouds and file 3 the program and matrices in use to calculate the extrinsic matrices from a set of corresponding points and one to estimate a best fit sphere. A correspondence search algorithm can be found in^21^ or provided by the authors upon reasonable request.
