# Supplementary figures and images for: Telecentric stereo 3D imaging with isotropic micrometer resolution bridges macro- and microscale in small Lepidopterans
Source: Sci Rep. 2025 Aug 6;15:28690. doi: 10.1038/s41598-025-13795-6 (PMC12329043; doi:10.1038/s41598-025-13795-6)

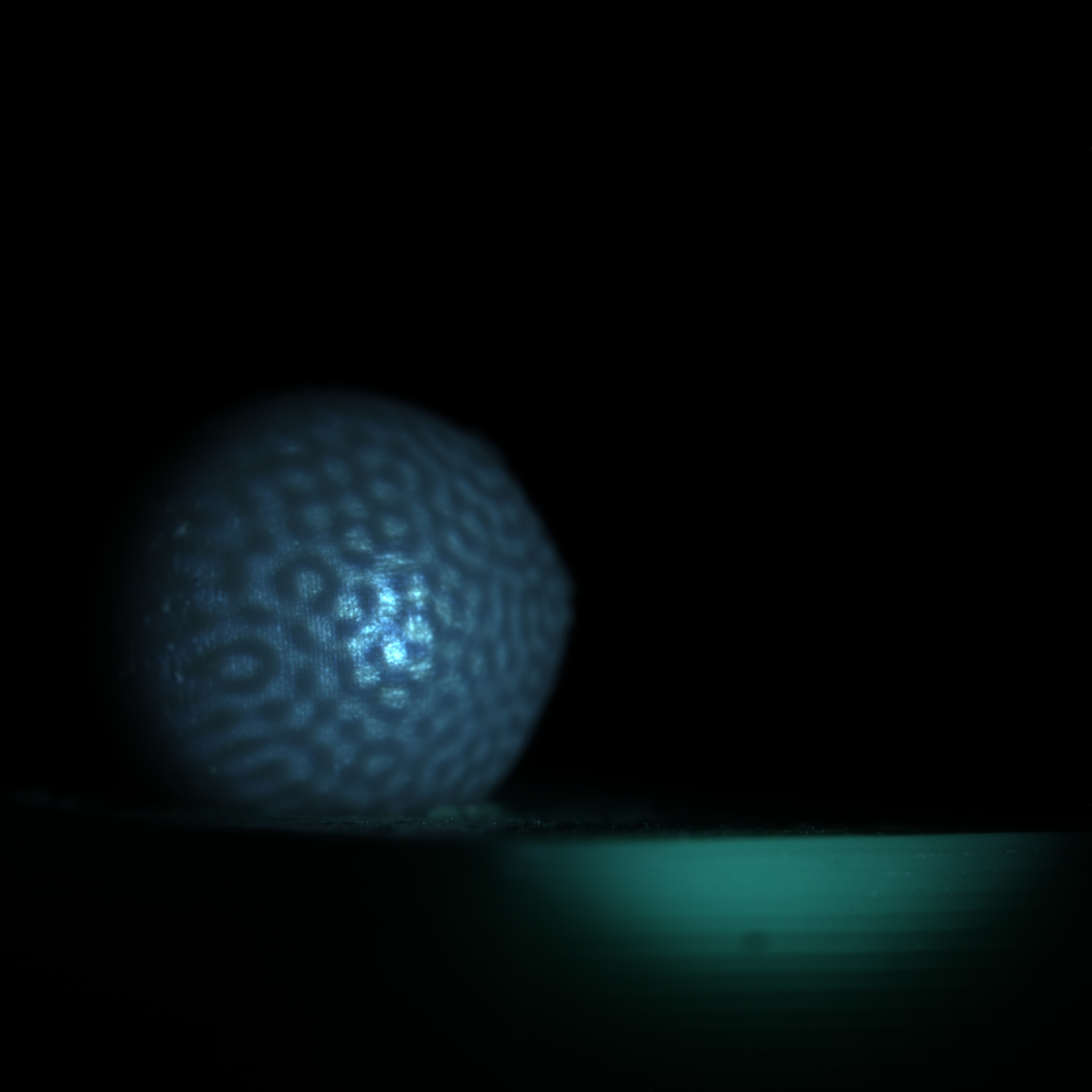

Supplement: Supplementary file 1 — Supplementary Information. [file 41598_2025_13795_MOESM1_ESM.zip › Additional_Material/Images_Sup_1/cam1_pos_0001_no_pattern.jpg]

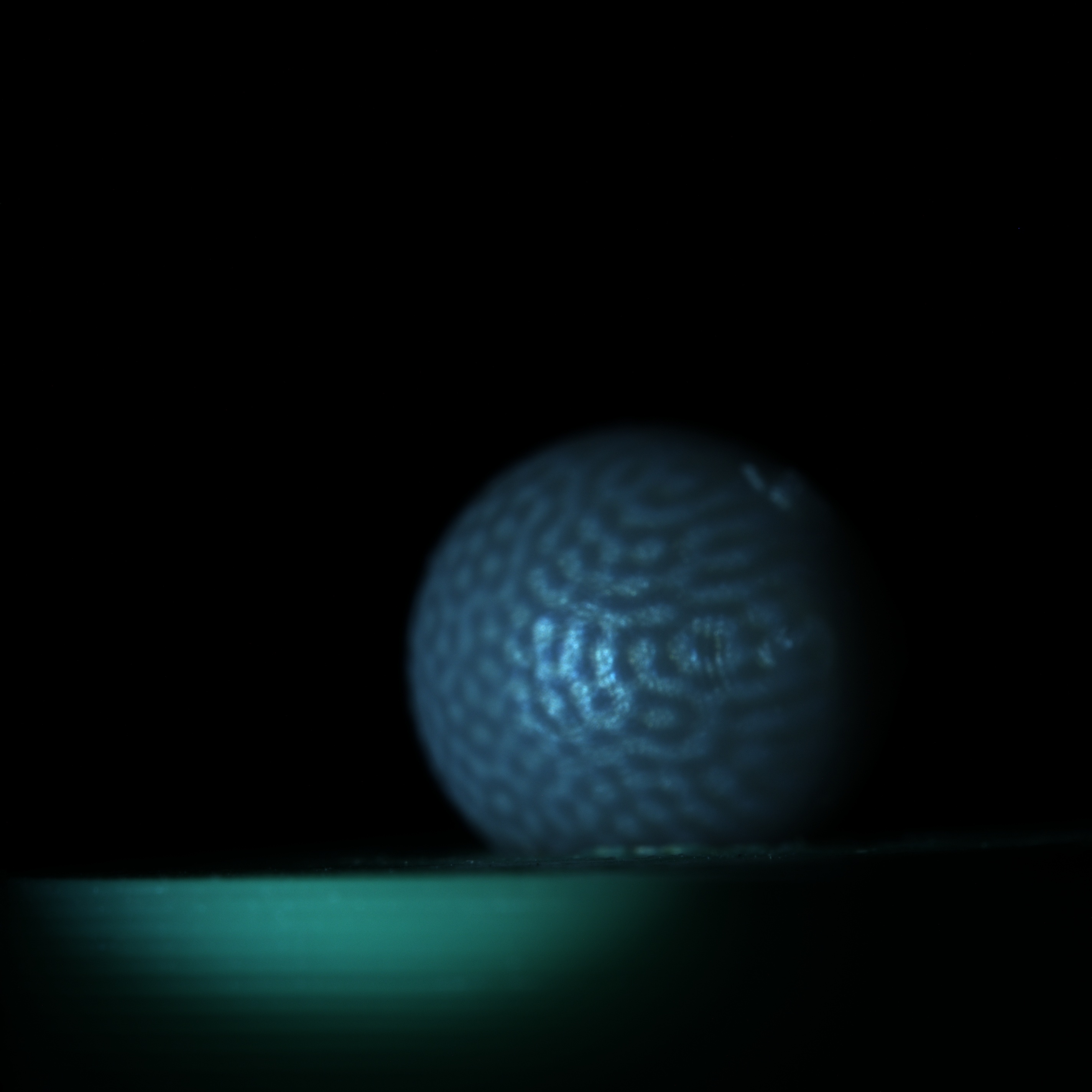

Supplement: Supplementary file 1 — Supplementary Information. [file 41598_2025_13795_MOESM1_ESM.zip › Additional_Material/Images_Sup_1/cam2_pos_0001_no_pattern.jpg]

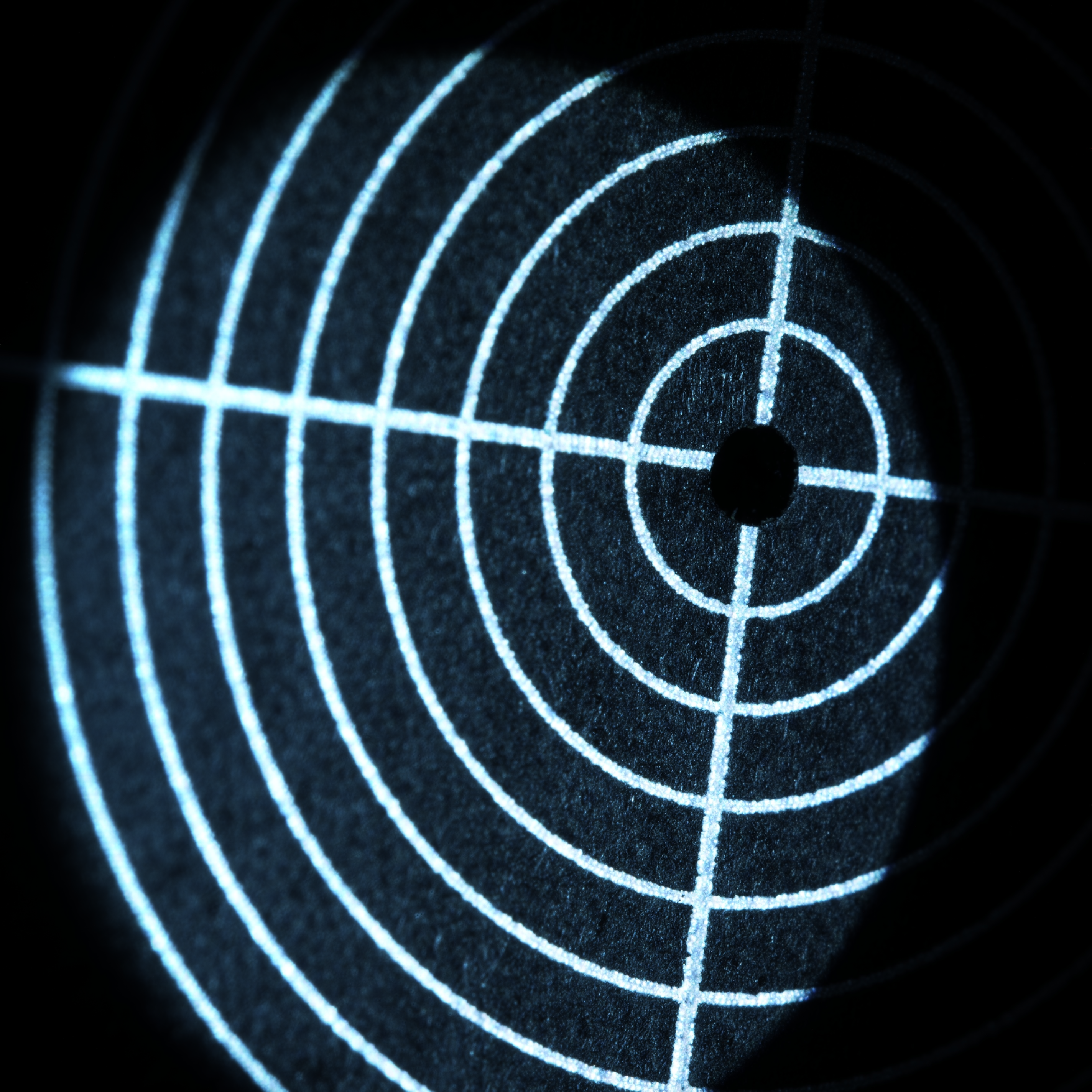

Supplement: Supplementary file 1 — Supplementary Information. [file 41598_2025_13795_MOESM1_ESM.zip › Additional_Material/Images_Sup_1/Lens Mount Alignment Plate.png]

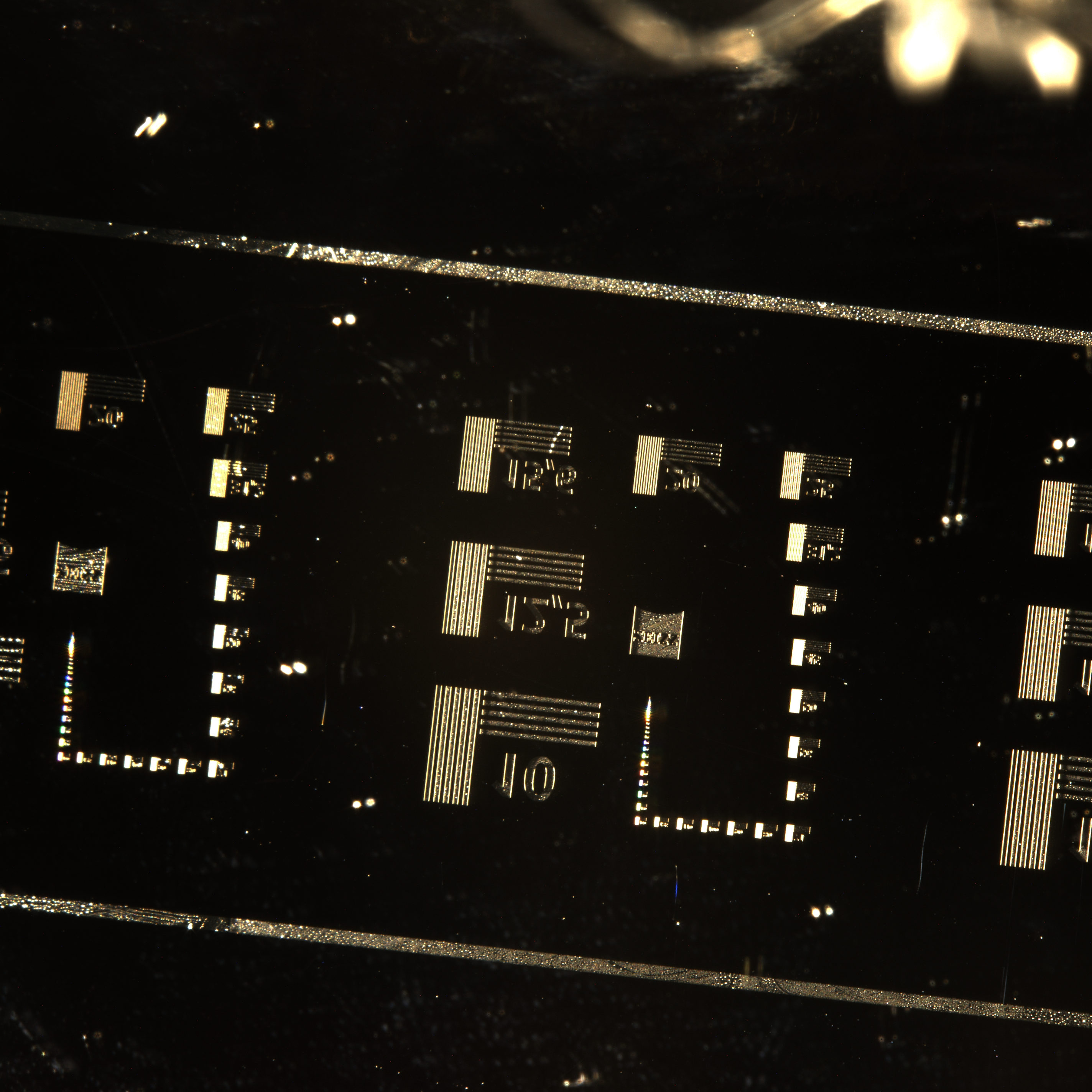

Supplement: Supplementary file 1 — Supplementary Information. [file 41598_2025_13795_MOESM1_ESM.zip › Additional_Material/Images_Sup_1/microscopic_test_chart.bmp]
